# Bowel Obstruction due to Migration of an Intragastric Balloon Necessitating Surgical Removal before Completion of the Recommended 6 Months

**DOI:** 10.1155/2012/414095

**Published:** 2012-10-03

**Authors:** Seyed Morteza Mousavi Naeini, Mahdi Sheikh

**Affiliations:** ^1^Department of General Surgery, Baghiatallah General Hospital, Baghiatallah University of Medical Sciences, Tehran, Iran; ^2^School of Medicine, Tehran University of Medical Sciences, Poursina Street, Tehran, Iran

## Abstract

We report a 25-year-old man with small bowel obstruction due to migration of a saline-filled intragastric balloon before the completion of the recommended 6 months of treatment who presented to the emergency department with abdominal pain. The patient had received a gastric balloon insertion 5 months prior. Within 24 hours of the original procedure, he noticed urine staining. The results of an endoscopy conducted the next day were normal. After ruling out other possible complications using endoscopy and confirming the diagnosis by computed tomography (CT) scan and conservative treatment for 48 hours the patient underwent surgery and the balloon was extracted. Due to the growing prevalence of obesity and the modalities used for treating it, physicians should be familiar with the side effects of each option and their presenting symptoms as well as the differential diagnosis they should not miss. Physicians must also improve their knowledge of how to approach these patients to avoid life-threatening complications caused by these modalities.

## 1. Introduction

Obesity is a major health problem challenging the modern world and is distributing insidiously throughout the globe. Because it is a major risk factor for many potential life-threatening conditions, different invasive and noninvasive therapeutic modalities are being used to help the individuals suffering from obesity return to a healthy life. Among these modalities, intragastric balloons are gaining popularity because of their efficacy, safety, and the ease of the method, as shown by some studies [1]. The balloon is placed endoscopically with sedation then inflated under direct endoscopic visualization with either air or saline and methylene blue. After several hours, the patient is discharged and followed up as an outpatient for the following 6 months. Then, the balloon is removed using the same method.

Most of the reported serious complications with the newer generations of balloons occur after 6 months of the placement of the balloon [2, 3]. Here, we report a case of small bowel obstruction due to leakage and deflation of a BioEnterics intragastric balloon (BIB) that migrated to the small bowel before completion of the 6 months recommended for the treatment with balloons. 

## 2. Case Report 

A 25-year-old man was brought to the emergency department with an 8-hour history of cramps and abdominal pain accompanied by anorexia and nausea. Since the beginning of his symptoms, he had passed one loose stool. His medical history was unremarkable except for an endoscopically inserted saline-filled intragastric balloon 5 months prior. On further investigation, it became clear that 24 hours after balloon placement the patient's symptoms (nausea and abdominal pain following balloon insertion) had suddenly disappeared and his urine had turned blue. The following day, endoscopy was repeated, and the gastric mucosa and balloon appeared normal without any signs of deflation or leakage.

The patient's initial body mass index (BMI) was 35.18, and during these 5 months he had lost 12 kilograms of his weight, reaching a BMI of 31.31.

On physical examination, he was in moderate distress and a state of mild dehydration. On abdominal palpation, he had diffuse tenderness in his epigastric and umbilical regions, though it was soft without any guarding or rebound and his bowel sounds were active. After admission, the patient began to present bilious vomiting and obstipation. An abdominal radiograph was taken, showing air fluid levels and small bowel distention concomitant with bowel obstruction. On the following day, the patient underwent upper gastrointestinal endoscopy in which gastroesophageal reflux disease type A (GERD-A) in the esophagus and reflux gastropathy in the stomach were observed and biopsied. No evidence of the balloon was found inside the stomach, confirming that it had migrated to the bowel.

**Figure 1:**
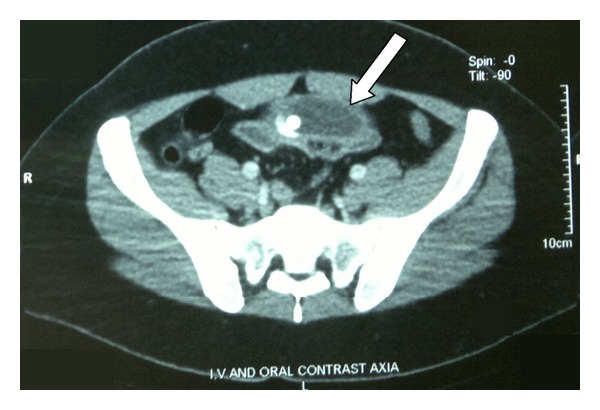
Abdominal CT scan showing the deflated intragastric balloon migrated to the small bowel (white arrow).

The initial treatment consisted of fluid resuscitation and nasogastric tube (NGT) insertion for bowel decompression. Oral and intravenous contrast computed tomography (CT) scans were performed and showed the balloon occluding the distal ileum (Figure 1). Proximal to the obstruction, dilated loops with air-fluid levels were visible, and distal to the obstruction, collapsed loops could be observed. In some images, intestinal wall thickening was also observed. After 48 hours of close observation, in hopes that the obstruction would resolve through conservative treatment, the patient was taken to the operation room, and laparotomy (6 cm mid-line incision) was performed. There was a small bowel obstruction due to migration of the balloon to a site 150 cm from the ileocecal valve. Enterotomy was performed, and the balloon was deflated and extracted. On the first postoperative day, the NGT was removed and on the second day, an oral diet began and the patient who had experienced an uneventful recovery was discharged. 

## 3. Discussion

Intragastric balloons were introduced in the early 1980s and have attracted the attention of physicians since their first use [7]. Later reports of serious side effects occurring at a relatively high rate accompanied by ineffectiveness for achieving and maintaining the desired weight [8, 9] led physicians and manufacturers to develop newer generations of gastric balloons that are technically improved and have reduced these potentially life-threatening complications. The newer generations of balloons are filled with saline to induce more effective weight loss and methylene blue to stain the urine in case of balloon rupture or deflation, facilitating early detection by the patient. 

After reviewing the literature, we noticed that less attention has been paid to this very important finding (urine discoloration), because only a minority of studies mention the presence of urine staining when reporting the complications [4–6] (Table 1).

In our case, the patient reported urine discoloration 24 hours after balloon placement. Despite the entirely normal endoscopy, the balloon deflated slowly over 5 months and migrated to the bowel, resulting in the need for surgery for its removal. This is a complication that could be easily avoided by changing the balloon or performing serial sonography (even in the presence of normal endoscopy) for early detection of balloon deflation or migration because ultrasound imaging is a safe and accurate method for assessing BioEnterics intragastric balloon (BIB) status [6].

Although many studies reported intragastric balloon placement as a safe method for weight loss, it was associated with a couple of complications in each study, for instance Lopez-Nava in his study which consisted of 714 patients reported an overall complication rate of 4.1% [10] or Genco in his study of 2515 patients reported an overall complication rate of 2.8% [1]. In our 16 years of experience with bariatric surgery, this was the second case of a potentially life-threatening complication of balloon treatment we encountered. The first was a case of gastric perforation due to balloon insertion which made the patient refer to the emergency department with severe abdominal pain within 24 hours of the procedure, both patients had the same symptoms and history. Due to the growing prevalence of obesity and the use of modalities such as intragastric balloons as well as their side effects, abdominal pain in an obese patient with a history of balloon insertion constitutes a diagnostic challenge because the majority of the reported complications, ranging from benign to life threatening, can present with abdominal pain and nausea or vomiting [5, 6, 10–16] (Table 2). Therefore, when the diagnosis is in doubt, performing an endoscopy and abdominal ultrasound imaging seems to be a reasonable step. This approach was used in our case because of the nonspecific clinical presentation and to rule out serious complications associated with balloon insertion.

After confirming the diagnosis of obstruction, if the patient is stable, conservative treatment and close observation may be a reasonable approach because sometimes the deflated balloon can pass the bowels without any further complication, as shown in some reports [5, 17]. If the obstruction has not resolved spontaneously after 48 hours, surgical removal of the balloon should be performed. In our case, as in most of the reported cases, the patient underwent a laparotomy for balloon extraction. Because bowel obstruction caused by intragastric balloons is relatively rare, most surgeons have not experienced a similar case and would therefore perform laparotomy to explore the abdominal cavity and extract the balloon. This operation can be performed laparoscopically by perforating the balloon and suctioning its contents. The balloon is then placed in a plastic bag for retrieval [2]. Using this method, we can help the patient avoid the complications of laparotomy and prevent the formation of lifetime scar.

## Figures and Tables

**Table 1:** Rate of balloon deflation and sensitivity of urine discoloration for detecting it.

Study	Balloons inserted *N*	Balloons deflated *N* (%)	Urine discoloration *N* (%)	Sensitivity of urine discoloration for balloon deflation
Loffredo et al. 2001 [4]	77	15 (19.4%)	9 (11.6%)	60%
Doldi et al. 2002 [5]	281	8 (2.8%)	5 (1.7%)	62.5%
Francica et al. 2004 [6]	131	18 (13.7%)	11 (8.3%)	61.1%

**Table 2:** Reported balloon complications presenting with abdominal pain.

Bowel obstruction	Gastric erosion
Gastric outlet obstruction	Esophagitis
Gastric perforation	Acute pancreatitis
Antral impaction	Acute cholecystitis
Gastric necrosis	Nonalcoholic steatohepatitis
Gastric dilatation	Hypokalemia
Gastric ulcer	Exacerbation of ulcerative colitis

## References

[1] Genco A, Bruni T, Doldi SB (2005). BioEnterics intragastric balloon: the Italian experience with 2,515 patients. *Obesity Surgery*.

[2] Vanden Eynden F, Urbain P (2001). Small intestine gastric balloon impaction treated by laparoscopic surgery. *Obesity Surgery*.

[3] Matar ZS, Mohamed AA, Abukhater M, Hussien M, Emran F, Bhat NA (2009). Small bowel obstruction due to air-filled intragastric balloon. *Obesity Surgery*.

[4] Loffredo A, Cappuccio M, De Luca M (2001). Three years experience with the new intragastric balloon, and a preoperative test for success with restrictive surgery. *Obesity Surgery*.

[5] Doldi SB, Micheletto G, Perrini MN, Librenti MC, Rella S (2002). Treatment of morbid obesity with intragastric balloon in association with diet. *Obesity Surgery*.

[6] Francica G, Giardiello C, Iodice G (2004). Ultrasound as the imaging method of choice for monitoring the intragastric balloon in obese patients: normal findings, pitfalls and diagnosis of complications. *Obesity Surgery*.

[7] Nieben OG, Harboe H (1982). Intragastric balloon as an artificial bezoar for treatment of obesity. *The Lancet*.

[8] Kramer FM, Stunkard AJ, Spiegel TA (1989). Limited weight losses with a gastric balloon. *Archives of Internal Medicine*.

[9] McFarland RJ, Grundy A, Gazet JC, Pilkington TRE (1987). The intragastric balloon: a novel idea proved ineffective. *British Journal of Surgery*.

[10] Lopez-Nava G, Rubio MA, Prados S (2011). BioEnterics; intragastric balloon (BIB). Single ambulatory center Spanish experience with 714 consecutive patients treated with one or two consecutive balloons. *Obesity Surgery*.

[11] Ubeda-Iglesias A, Irles-Rocamora JA, Povis-Lopez CD (2012). Antral impaction and cardiorespiratory arrest. Complications of the intragastric balloon. *Medicina Intensiva*.

[12] Pretolesi F, Redaelli G, Papagni L, Derchi LE (2001). Intragastric balloon for morbid obesity causing chronic gastric dilatation. *European Radiology*.

[13] Forlano R, Ippolito AM, Iacobellis A (2010). Effect of the BioEnterics intragastric balloon on weight, insulin resistance, and liver steatosis in obese patients. *Gastrointestinal Endoscopy*.

[14] Mohammed AE, Benmousa A (2008). Acute pancreatitis complicating intragastric balloon insertion. *Case Reports in Gastroenterology*.

[15] Benchimol AK, Cardoso IS, Fandiño J, Bittar T, Freitas S, Coutinho WF (2007). Non-alcoholic steatohepatitis induced by fast weight loss during the use of intragastric balloon—a case report. *Arquivos Brasileiros de Endocrinologia e Metabologia*.

[16] Nikolic M, Mirosevic G, Ljubicic N (2011). Obesity treatment using a bioenterics intragastric balloon (BIB)—preliminary croatian results. *Obesity Surgery*.

[17] Conti PS, Warner CH, Fleisher AG, Nay HR, Jones B (1988). Bowel obstruction caused by gastric balloons. *American Journal of Roentgenology*.

